# hUC-MSCs regulating the balance of Th17/Treg in Henoch Schönlein purpura via STAT3 pathway

**DOI:** 10.1016/j.jped.2026.101613

**Published:** 2026-10-13

**Authors:** Li Zhang, Tao Chen, Jieqiong Zeng, Anna Qiao, Bingjie Zhou, Xianjuan Shen, Zhiyuan Tang, Jianmei Zhao

**Affiliations:** a School of Medicine, Nantong University, Nantong, China; b The Second Affiliated Hospital of Soochow University, Suzhou, China; c Department of Pediatrics, Affiliated Hospital of Nantong University, Nantong, China; d Department of Laboratory, Affiliated Hospital of Nantong University, Nantong, China; e Department of Pharmacy, Affiliated Hospital of Nantong University, Nantong, China

**Keywords:** Henoch-Schonlein purpura, Human umbilical cord mesenchymal stem cells, Stat3 pathway, Th17/Treg

## Abstract

**Objective:**

This study explores the relationship between T helper 17 (Th17)/regulatory T (Treg) cell balance, human umbilical cord mesenchymal stem cells (hUC-MSCs), and immune dysregulation in Henoch-Schönlein purpura (HSP).

**Methods:**

Peripheral blood and clinical data were collected from children with acute HSP and healthy controls. Th17/Treg-related transcription factors and cytokines were measured by reverse transcription quantitative polymerase chain reaction (RT-qPCR) and enzyme-linked immunosorbent assay (ELISA). *In vitro*, human umbilical vein endothelial cells were stimulated with HSP serum and co-cultured with hUC-MSCs, with or without the signal transducer and activator of transcription 3 (STAT3) agonist colivelin TFA. STAT3 signaling, interleukin-6 (IL-6) expression, and endothelial migration were analyzed. *In vivo*, an ovalbumin-induced HSP rat model was treated with hUC-MSCs or stem cells plus colivelin TFA, followed by evaluation of pathological injury, STAT3 activation, and splenic Th17/Treg ratios.

**Results:**

Children with HSP exhibited significant Th17/Treg imbalance, with elevated RORγt and IL-17 and decreased FOXP3 and IL-10 (P < 0.05). *In vitro*, stem cells suppressed IL-6/STAT3 signaling, reduced STAT3 phosphorylation, and promoted endothelial repair, whereas colivelin TFA abolished these effects. In the rat model, stem cell transplantation alleviated inflammation in skin, kidney, joint, and intestinal tissues by downregulating the IL-6/STAT3/RORγt axis and restoring Th17/Treg balance; these benefits were negated by colivelin TFA.

**Conclusion:**

Th17/Treg imbalance is an immunopathological feature of HSP. hUC-MSCs restore immune homeostasis by inhibiting STAT3 signaling, highlighting STAT3 as a potential therapeutic target and supporting further investigation of stem cell-based therapy.

## Introduction

Henoch-Schönlein purpura (HSP), also known as immunoglobulin A vasculitis, is the most common systemic small-vessel vasculitis in children [1]. It presents clinically with non-thrombocytopenic purpura, often accompanied by arthralgia, abdominal pain, gastrointestinal bleeding, hematuria, and proteinuria [2]. Severe cases may progress to nephritis characterized by IgA deposition in glomerular mesangium and immune-mediated injury [3,4]. Despite well-documented clinical features, the immunopathogenesis of HSP remains incompletely understood.

The balance between T helper 17 (Th17) cells and regulatory T cells (Tregs) plays a central role in maintaining immune homeostasis [5]. Disruption of this balance promotes excessive inflammatory responses, autoimmunity, and tissue damage. Increasing evidence implicates a Th17/Treg imbalance in the pathogenesis of HSP [6]. Among signaling pathways involved, STAT3 is a critical regulator of T-cell differentiation, particularly in promoting Th17 expansion and inhibiting Treg stability [7]. Aberrant activation of STAT3 amplifies inflammation and dysregulates the Th17/Treg balance [8] suggesting that modulation of this pathway may have therapeutic value in HSP.

Mesenchymal stem cells (MSCs) possess immunomodulatory, regenerative, and anti-inflammatory properties, showing therapeutic potential in autoimmune and vascular diseases [9,10]. Human umbilical cord-derived MSCs (hUC-MSCs) offer advantages of low immunogenicity, strong proliferative capacity, and accessibility. Previous studies demonstrate that MSCs regulate Th17/Treg balance [11], but whether hUC-MSCs restore this balance in HSP through STAT3 inhibition remains unclear.

To address this, the authors integrated clinical data from HSP patients, *in vitro* endothelial functional assays, and an established ovalbumin-induced rat model of HSP, employing the STAT3-specific agonist colivelin TFA to mechanistically dissect the pathway's role. The present results delineate a mechanism through which hUC-MSCs restore immune homeostasis and delineate the suppression of the IL-6/STAT3/RORγt axis as a pivotal mechanism, underscoring the pathway's therapeutic relevance in HSP.

## Methods

### Patient blood samples and ethical considerations

Peripheral blood samples were collected from 42 children diagnosed with HSP at the Department of Pediatrics, Affiliated Hospital of Nantong University, between June 2023 and December 2024. Samples from 42 age-matched healthy children served as controls. Diagnosis of HSP was based on the 2010 EULAR/PRINTO/PRES criteria [12]. Healthy controls had no recent infections (within 4 weeks), immunodeficiency, genetic disorders, or major systemic diseases. Written informed consent was obtained from legal guardians. The study was approved by the Ethics Committee of the Affiliated Hospital of Nantong University (Approval No.: 2022-K025-01). All patients received standard symptomatic and supportive treatments; severe cases with organ involvement received corticosteroids. Long-term progression to renal failure was not evaluated in this cross-sectional cohort.

### Cell culture

Human umbilical vein endothelial cells (HUVECs) and human umbilical cord mesenchymal cells (hUC-MSCs) were obtained from the Department of Obstetrics and Gynecology, Affiliated Hospital of Nantong University. hUC-MSCs had been authenticated for characteristic phenotypic and differentiation potential [13].

HUVECs were cultured in DMEM/F12 medium with 10% heat-inactivated serum from healthy children (Ctrl) or from HSP patients (HSP). After 24 h of stimulation, HSP-conditioned HUVECs were co-cultured with hUC-MSCs using a Transwell system equipped with a 0.4-μm pore size polyester membrane insert (BD Biosciences, California, USA), allowing paracrine interaction without direct cell–cell contact (HSP+MSC). To investigate the involvement of STAT3 signaling, HUVECs were pretreated with colivelin TFA (MCE, New Jersey, USA) at an optimized concentration for 1 h prior to co-culture (HSP+MSC+TFA).

For immune cell experiments, human CD4⁺ T cells were isolated from peripheral blood mononuclear cells (PBMCs) of healthy donors using magnetic-activated cell sorting (MACS) with a CD4⁺ T cell isolation kit (Miltenyi Biotec, California, USA), according to the manufacturer’s protocol. Purified CD4⁺ T cells were cultured in RPMI-1640 medium supplemented with 10% heat-inactivated human serum. Cells were then exposed to Ctrl or HSP serum for 24 h, followed by co-culture with hUC-MSCs using the same Transwell system described above. Where indicated, CD4⁺ T cells were treated with colivelin TFA for 1 h prior to co-culture.

To assess STAT3 signaling responses in hUC-MSCs, hUC-MSCs were cultured alone and incubated with Ctrl serum, HSP serum, or HSP serum in the presence of colivelin TFA for 24 h. Cells were then harvested for protein extraction and subsequent analysis of IL-6, STAT3, and p-STAT3 expression.

### HSP animal model

Forty 4-week-old male Sprague-Dawley rats were obtained from the Standardized Experimental Animal Breeding Base, Nantong University. An HSP animal model was induced using ovalbumin (OVA, Solarbio, Beijing, China) emulsion and saline injections [14]. Rats were randomly assigned to four groups (n = 10/group): Healthy control (Ctrl), HSP model (HSP), hUC-MSCs treatment (HSP+MSC), and hUC-MSCs + colivelinTFA treatment (HSP+MSC+TFA). Groups HSP, HSP+MSC, and HSP+MSC+TFA received intraperitoneal OVA emulsion (10 mg/kg) weekly for 3 weeks, followed by OVA challenge via tail vein and intradermal injections. The HSP+MSC group additionally received hUC-MSCs (2 × 10^6^ cells/mL, 1 mL, via tail vein) every 3 days after hypersensitivity induction. The HSP+MSC+TFA group also received colivelin TFA (0.1 mg/mL, 10 mL/kg, intraperitoneally) after each hUC-MSCs injection (Supplementary 1A). The dosing regimen was selected based on preliminary experiments and supported by previously published studies [15]. At the end of modeling, rats were euthanized, and kidney, joint, and intestinal tissues were collected. All procedures were approved by the Animal Welfare Ethics Committee (Approval No.: S20230321-002).

### RT-qPCR

Peripheral blood mononuclear cells (PBMCs) were isolated by Ficoll density gradient centrifugation (Lymphoprep, Thermo Fisher, USA). Total RNA was extracted using Trizol reagent, and reverse transcription was performed with the PrimeScript™ RT Reagent Kit (TaKaRa, Cat #RR037A, Kusatsu, Japan). Quantitative PCR was conducted using SYBR™ Green Master Mix (Applied Biosystems, Shanghai, China) with 40 amplification cycles. GAPDH served as the internal control. Primer sequences are listed in Table 1.

**Table 1:** Primer sequences.

Gene	Organism	Forward Primer	Reverse Primer
GAPDH	Homo sapiens	TGATGACATCAAGAAGGTGGTGAA	TCCTTGGAGGCCATGTGGGCCAT
Gapdh	*Rattus norvegicus*	GACATGCCGCCTGGAGAAAC	AGCCCAGGATGCCCTTTAGT
RORC	Homo sapiens	AGAAGACCCACACCTCACAA	GGTTCCTGTTGCTGCTGTTG
Rorc	*Rattus norvegicus*	AACATCTCGGGAGTTGCTGG	GCTGAAGTCCACGTCTTCCA
FOXP3 Foxp3	Homo sapiens *Rattus norvegicus*	CCCTGACAGCCTGTTTGC	TGGCGTAGGTGAAAGGGG
IL6	Homo sapiens	CACTGGCAGAAAACAACCTG	TGTACTCATCTGCACAGCTCT
Il6	*Rattus norvegicus*	CACTTCACAAGTCGGAGGCT	AGCACACTAGGTTTGCCGAG
STAT3	Homo sapiens	CTGGCCCCTTGGATTGAGAG	CTGTTCTAGATCCTGGGCCG
Stat3	*Rattus norvegicus*	CAACGACCTGCAGCAATACC	CCATGTCAAACGTGAGCGAC

### Enzyme-linked immunosorbent assay (ELISA)

Serum concentrations of IL-17 and IL-10 were quantified using commercial human ELISA kits (IL-17A and IL-10, Thermo Fisher Scientific, California, USA). Serum samples were diluted 1:4 in the provided assay diluent. A standard curve was generated using the recombinant cytokine standard (range: 0–400 pg/mL). Briefly, 100 µL of diluted samples or standards were added to pre-coated 96-well plates and incubated for 2 h at room temperature on a plate shaker (300 rpm). After four washes, 90 µL of detection antibody was added per well and incubated for 1 h. Following another wash step, streptavidin-HRP was added, incubated for 30 min in the dark, and the plate was washed again. TMB substrate was added, incubated for 15 min in the dark, and the reaction was stopped with 50 µL of stop solution per well. Absorbance was measured at 450 nm with a 570 nm reference using a microplate reader. Cytokine concentrations were interpolated from the standard curve via a 4-parameter logistic (4PL) regression model.

### Hematoxylin–eosin (H&E) staining

Tissue samples (kidney, joint, and intestine) were fixed in 4% paraformaldehyde (in 0.1 M phosphate buffer, pH 7.4) at 4 °C for 24 h, washed, and processed through graded ethanol, xylene, and paraffin wax under standard protocols. Sections (4 μm) were cut using a rotary microtome (Leica RM2235, Wetzlar, Germany), mounted on coated slides, dried at 37 °C overnight, and baked at 55 °C for 2 h before staining. For H&E staining, sections were deparaffinized in xylene (two 10-min incubations; Sinopharm AR grade, Sigma-Aldrich ACS grade) and rehydrated through a graded ethanol series (100%, 90%, 80%, 70%; 5 min each, Fisher Scientific HPLC grade). After rinsing in deionized water, nuclei were stained with Harris hematoxylin solution from a commercial H&E staining kit (Beyotime Biotechnology, Shanghai, China) for 5 min, rinsed, and cytoplasm was counterstained with the accompanying eosin Y solution (1 s). Staining quality was verified microscopically after each step.

Sections were dehydrated through an ascending ethanol series (70% to 100%; 5 min each), cleared in xylene (two 15-min changes), and mounted with neutral balsam. Slides were examined under a light microscope, and representative images were captured digitally for analysis.

### Western blot

Total protein was extracted with RIPA buffer, separated by SDS-PAGE, and transferred to PVDF membranes. Membranes were blocked and incubated with primary antibodies against IL-6 (Proteintech, Wuhan, China, 1:1000), RORγt (Affinity Biosciences, Cincinnati, USA, 1:1000), STAT3 (Proteintech, Wuhan, China, 1:1000), p-STAT3 (Proteintech, Wuhan, China, 1:1000), and GAPDH (Proteintech, Wuhan, China, 1:10000), followed by HRP-conjugated secondary antibodies (Sangong Biotech, Shanghai, China, 1:10000). Bands were visualized and quantified. Each experiment was independently repeated at least three times.

### Flow cytometry

PBMCs and rat splenocytes were isolated, red blood cells lysed, and lymphocytes enriched. Cells were surface-stained with FITC-conjugated anti-human CD4 antibody (Biolegend, California, USA) or PerCP/Cy5.5-conjugated anti-rat CD4 antibody (Biolegend, California, USA) combined with Fc block, and stimulated for 4 h at 37°C (with Leukocyte Activation Cocktail, BD, New Jersey, USA). For intracellular staining, cells were fixed, permeabilized, and stained with APC-conjugated anti-FOXP3 (Biolegend, California, USA) and PE-conjugated anti-IL-17A (Biolegend, California, USA) for 30 min in the dark. After washing, cells were resuspended in PBS, filtered, and analyzed on a BD FACS Aria III flow cytometer. Data were acquired on a BD FACS Aria III cytometer and analyzed with FlowJo v10.8 software.

### Cell viability assay (Cell counting kit-8, CCK-8)

Colivelin TFA was serially diluted in basal culture medium to concentrations of 0, 2.5, 5, 7.5, 10, and 12.5 mM. HUVECs were harvested using 0.25% trypsin-EDTA, resuspended in complete medium, and seeded into a 96-well plate at a density of 5 × 10³ cells/well (100 µL/well). After overnight incubation (37°C, 5% CO2), the medium was replaced with 100 µL of fresh medium containing the corresponding colivelin TFA concentration. Following a 24-h treatment under standard conditions, 10 µL of CCK-8 reagent (Dojindo, Kamimashiki-gun, Japan) was added to each well. The plate was protected from light and incubated at 37 °C for 3–4 h. Absorbance at 450 nm was measured using a microplate reader. Each concentration was tested in quadruplicate or quintuplicate, and blank control wells (medium only) were included for background subtraction. The mean optical density (OD) of replicates was used for analysis.

### Wound healing assay

HUVECs were seeded in 6-well plates at 5 × 105 cells/well and cultured until 90% confluent. A standardized cross-shaped wound was created using a sterile 200 µL pipette tip (orifice diameter: 0.4 mm) by dragging the tip vertically and horizontally across the monolayer to generate two perpendicular scratches spaced 1 mm apart. Wounded monolayers were washed three times with pre-warmed PBS, then incubated in 2 mL of serum-free DMEM/F12 medium supplemented with 1% penicillin–streptomycin. Phase-contrast images of the initial wound (0 h) were captured at 200 × magnification using an inverted light microscope. After 24 ± 0.5 h incubation under standard conditions (37 °C, 5% CO₂), cells were fixed with 4% paraformaldehyde and stained with 0.1% (w/v) crystal violet. The wound area at 24 h was imaged under identical settings and quantified using ImageJ software.

### Statistical analysis

Data are presented as mean ± SEM or mean ± SD from at least three independent experiments. Normality of continuous variables was assessed using the Shapiro-Wilk test (all P > 0.05), Two-group comparisons were performed with Student's *t*-test, and multi-group comparisons with one-way ANOVA followed by post hoc testing. A p-value < 0.05 was considered statistically significant.

## Results

### Th17/Treg imbalance in children with HSP

The authors analyzed PBMCs from 42 pediatric HSP patients and 42 healthy controls (demographic characteristics in Table 2; no baseline differences between groups). Flow cytometry revealed a significant increase in Th17 cells and a reduction in Tregs among CD4^+^ T cells from HSP patients (Figure 1A-E). Consequently, the Th17/Treg ratio was markedly elevated in the HSP group (Figure 1F). Consistent with these findings, RT-qPCR showed increased expression of the Th17 transcription factor RORC and decreased expression of the Treg transcription factor FOXP3 in patient PBMCs (Figure 1G-H). Cytokine profiling further confirmed this imbalance: serum IL-17 was elevated, while IL-10 was reduced in HSP patients compared with controls (Figure 1I-J).

**Table 2:** Baseline clinical data of each group.

Characteristics	Control (n = 42)^1^	HSP (n = 42)^1^	P value
Gender			0.13*
Male	24(57%)	17(40%)	
Female	18(43%)	25(60%)	
Age (years)	7.09(4.24)	8.08(2.80)	0.21#
Height (cm)	124.2(37.2)	137.72(18.34)	0.13#
Weight (kg)	31.32(19.77)	30.03(11.06)	0.71#

1 N(%); Mean (SD).

⁎ Pearson's Chi-squared test.

# Student's *t*-test.

**Figure 1:**
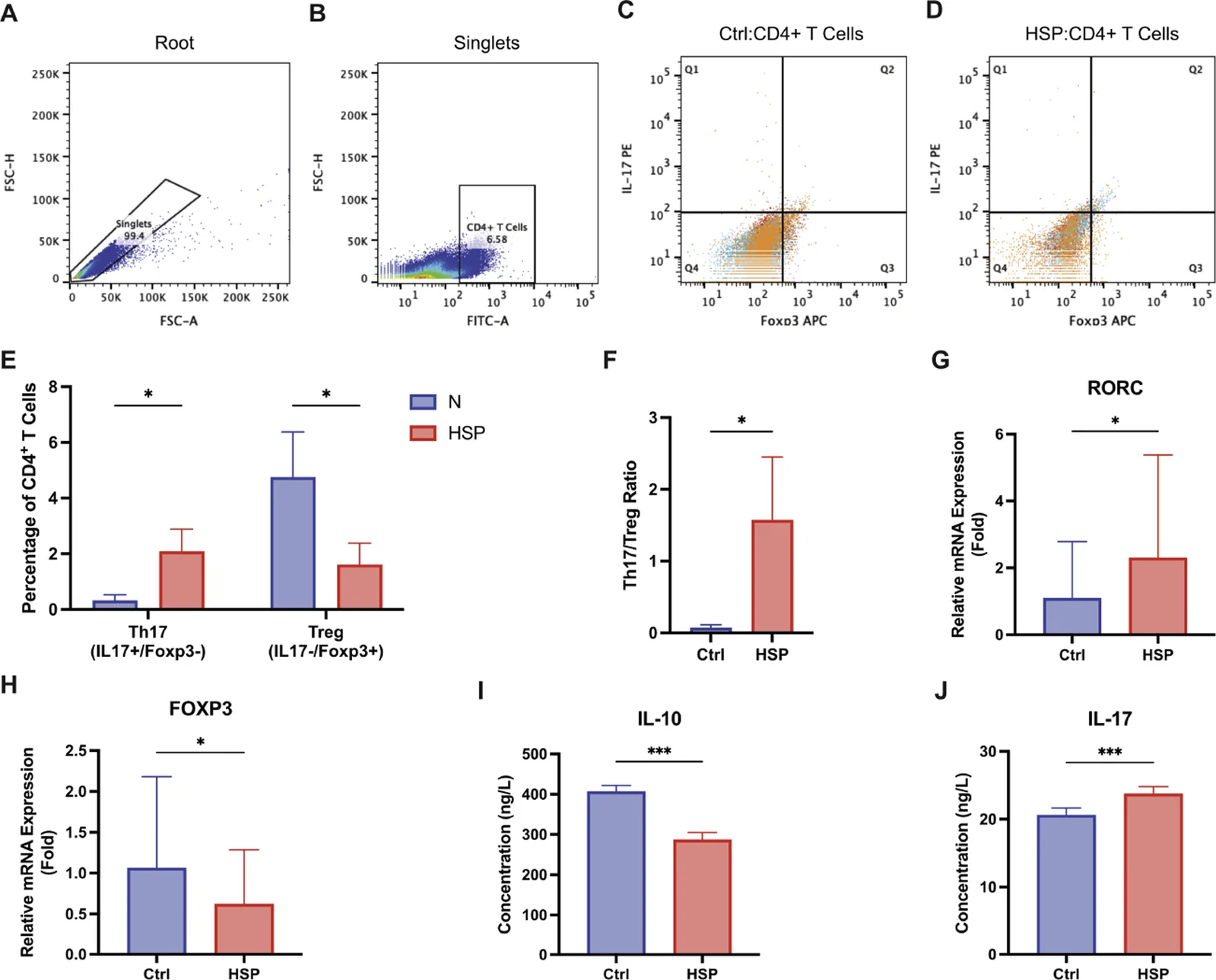
Th17/Treg imbalance in HSP children. Flow cytometry and molecular analysis of Th17 and Treg subsets in PBMCs from healthy controls (Ctrl, n = 42) and HSP patients (HSP, n = 42). (A–B) Representative flow cytometry gating strategy from one subject. (A) Singlets gate based on FSC-A/FSC—H scatter plot. (B) CD4⁺ T cells gate (FITC-A⁺, 99.1% of singlets). (C-D) Identification of Th17 (CD4⁺IL-17A⁺, upper right quadrant) and Treg (CD4⁺FOXP3⁺, lower right quadrant) subsets. (E) Percentage of Th17 and Treg cells among CD4^+^ T cells (n = 42/group). (F) Th17/Treg ratio. (G–H) Relative mRNA expression of RORC and FOXP3 (n = 42/group). (I–J) Serum concentrations of IL-10 and IL-17 (n = 42/group). Data are means ± SD, * P < 0.05; *** P < 0.001.

### hUC-MSCs ameliorate HSP-induced endothelial dysfunction

HUVECs cultured with HSP serum displayed abnormal morphology and impaired wound closure, consistent with severe endothelial dysfunction (Figure 2A–B). Co-culture with hUC-MSCs restored normal cell morphology and significantly enhanced migration (Figure 2A–C). However, STAT3 reactivation with colivelinTFA abolished these benefits, reinstating both morphological abnormalities and defective wound healing.

**Figure 2:**
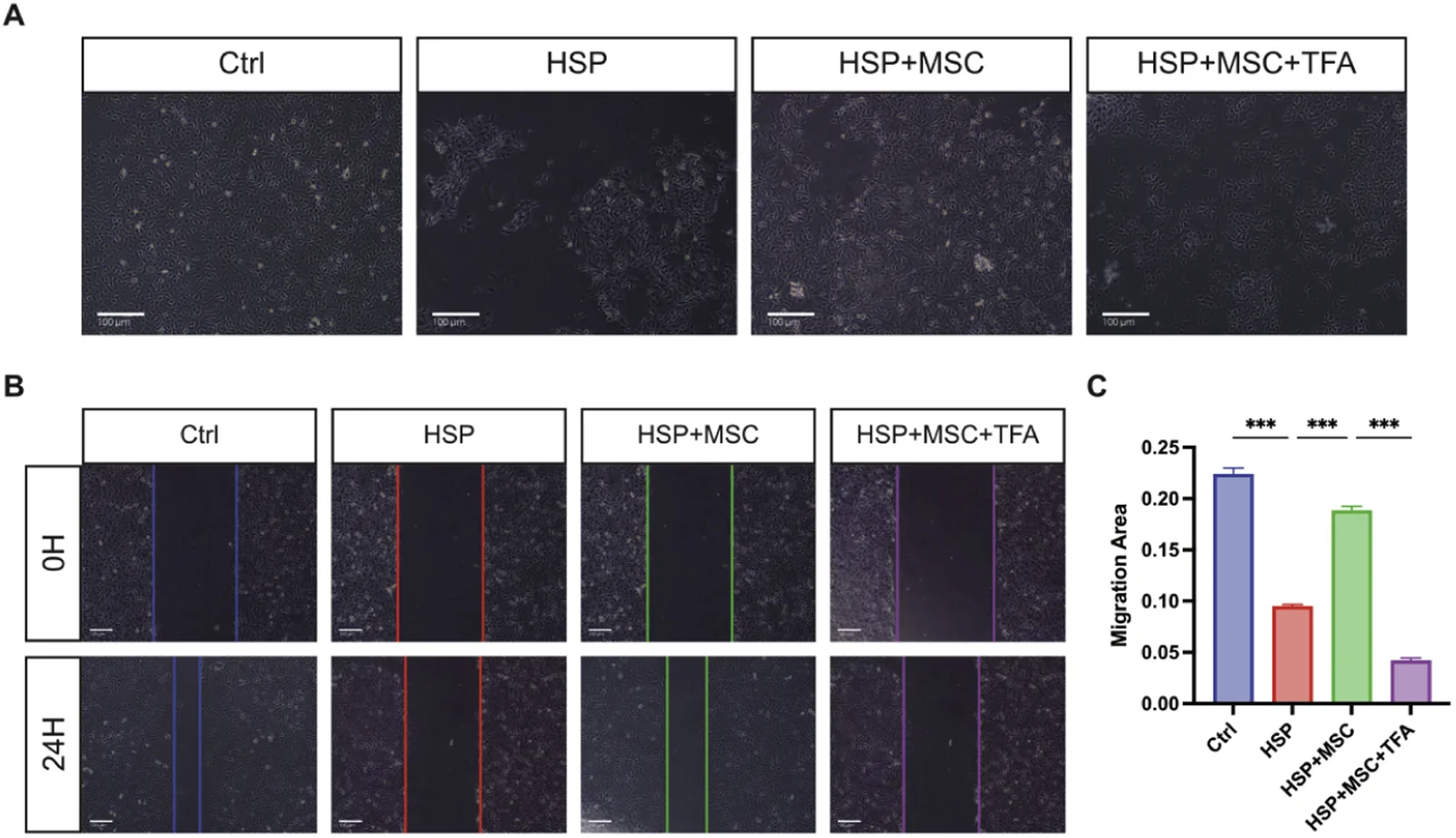
hUC-MSCs restore endothelial morphology and migration by suppressing STAT3. (A) Representative images of HUVECs cultured under the indicated conditions. Scale bar = 100 µm. (B) Wound healing assay images at 0 h and 24 h. Scale bar = 100 µm. (C) Quantification of wound closure. Ctrl: HUVECs cultured with 10% heat-inactivated serum from healthy children. HSP: HUVECs cultured with 10% heat-inactivated serum from HSP patients. HSP + MSC: HSP-conditioned HUVECs co-cultured with hUC-MSCs. HSP+MSC+TFA: HUVECs pretreated with colivelin TFA before HSP-conditioned and co-culture. Data are mean ± SEM, n = 3 independent experiments. *** P < 0.001.

### hUC-MSCs suppress STAT3 activation in an HSP cellular model

Exposure of HUVECs to HSP serum resulted in a marked increase in IL6 and STAT3 mRNA expression (Figure 3B–C), accompanied by elevated protein levels of IL-6, total STAT3, and phosphorylated STAT3 (p-STAT3) (Figure 3D–G), compared with control serum–treated cells. Co-culture with hUC-MSCs significantly reduced IL-6 and p-STAT3 protein levels in HSP-stimulated HUVECs, while total STAT3 expression was only modestly affected.

**Figure 3:**
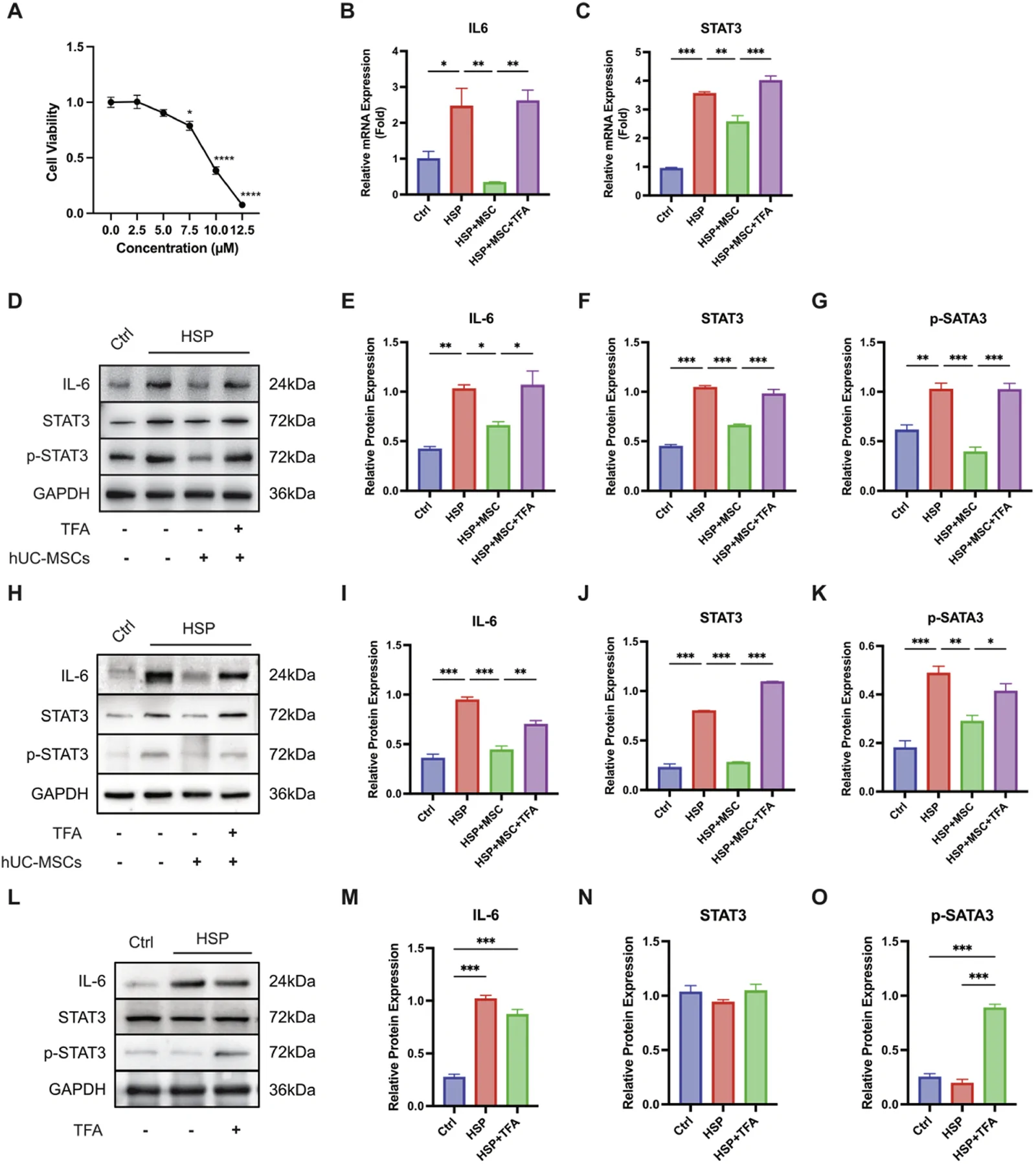
hUC-MSCs inhibit STAT3 activation in HSP-stimulated endothelial and immune cells. (A) CCK-8 assay assessing colivelin TFA cytotoxicity in HUVECs, identifying 5.0 μM as a non-cytotoxic working concentration. (B-C) Relative mRNA expression of IL6 and STAT3 in HUVECs exposed to control serum (Ctrl), HSP serum (HSP), HSP serum co-cultured with hUC-MSCs (HSP+MSC), or HSP serum co-cultured with hUC-MSCs and colivelin TFA (HSP+MSC+TFA). (D) Western blot analysis of IL-6, STAT3, and phosphorylated STAT3 (p-STAT3) in HUVECs; GAPDH served as a loading control. (E-G) Quantification of corresponding protein levels. (H) Western blots analysis of IL-6, STAT3, and p-STAT3 in CD4^+^ T cells under the indicated treatment conditions. (I-J) Quantification of protein expression in CD4^+^ T cells. (L) Western blot analysis of IL-6, STAT3, and p-STAT3 in hUC-MSCs cultured with Ctrl serum, HSP serum, or HSP serum plus colivelin TFA. (M-O) Quantification of protein expression in hUC-MSCs. Data are mean ± SEM; n = 3 independent experiments. * P < 0.05, ** P < 0.01, *** P < 0.001.

To assess the effect of pharmacological STAT3 modulation, colivelin TFA was applied as a STAT3 agonist. CCK-8 assays showed that colivelin TFA at 5.0 μM did not affect HUVEC viability and was therefore used for subsequent experiments (Figure 3A). Under these conditions, colivelin TFA treatment increased IL-6 and p-STAT3 expression in HSP serum–stimulated HUVECs co-cultured with hUC-MSCs, restoring protein levels comparable to those observed in HSP-treated cells (Figure 3B–G).

Similar expression patterns were observed in CD4⁺ T cells. HSP stimulation increased IL-6, total STAT3, and p-STAT3 protein levels, whereas co-culture with hUC-MSCs reduced the abundance of these proteins (Figure 3H–J). Addition of colivelin TFA reversed the hUC-MSC–associated reductions in IL-6 and p-STAT3 expression in CD4⁺ T cells.

In hUC-MSCs, exposure to HSP serum increased IL-6 protein expression, while no significant changes were detected in total STAT3 or p-STAT3 levels (Figure 3L–O). Treatment with colivelin TFA further increased IL-6 and p-STAT3 expression in hUC-MSCs without altering STAT3 abundance.

### Systemic administration of hUC-MSCs reduces multi-organ damage in a murine HSP model

To investigate the therapeutic potential of hUC-MSCs *in vivo*, the authors established an ovalbumin (OVA) induced HSP rat model. Compared with controls (Supplementary 1B), HSP rats developed widespread purpuric lesions (Supplementary 1C) and histological evidence of multi-organ injury, including renal mesangial proliferation, joint edema, and intestinal villous atrophy (Figure 4). Systemic administration of hUC-MSCs markedly alleviated these abnormalities, reducing skin lesions (Supplementary 1D) and restoring renal, joint, and intestinal architecture (Figure 4). In contrast, co-treatment with colivelin TFA abrogated the therapeutic effects of hUC-MSCs, with rats showing persistent skin lesions, lymphadenopathy, and severe tissue pathology.

**Figure 4:**
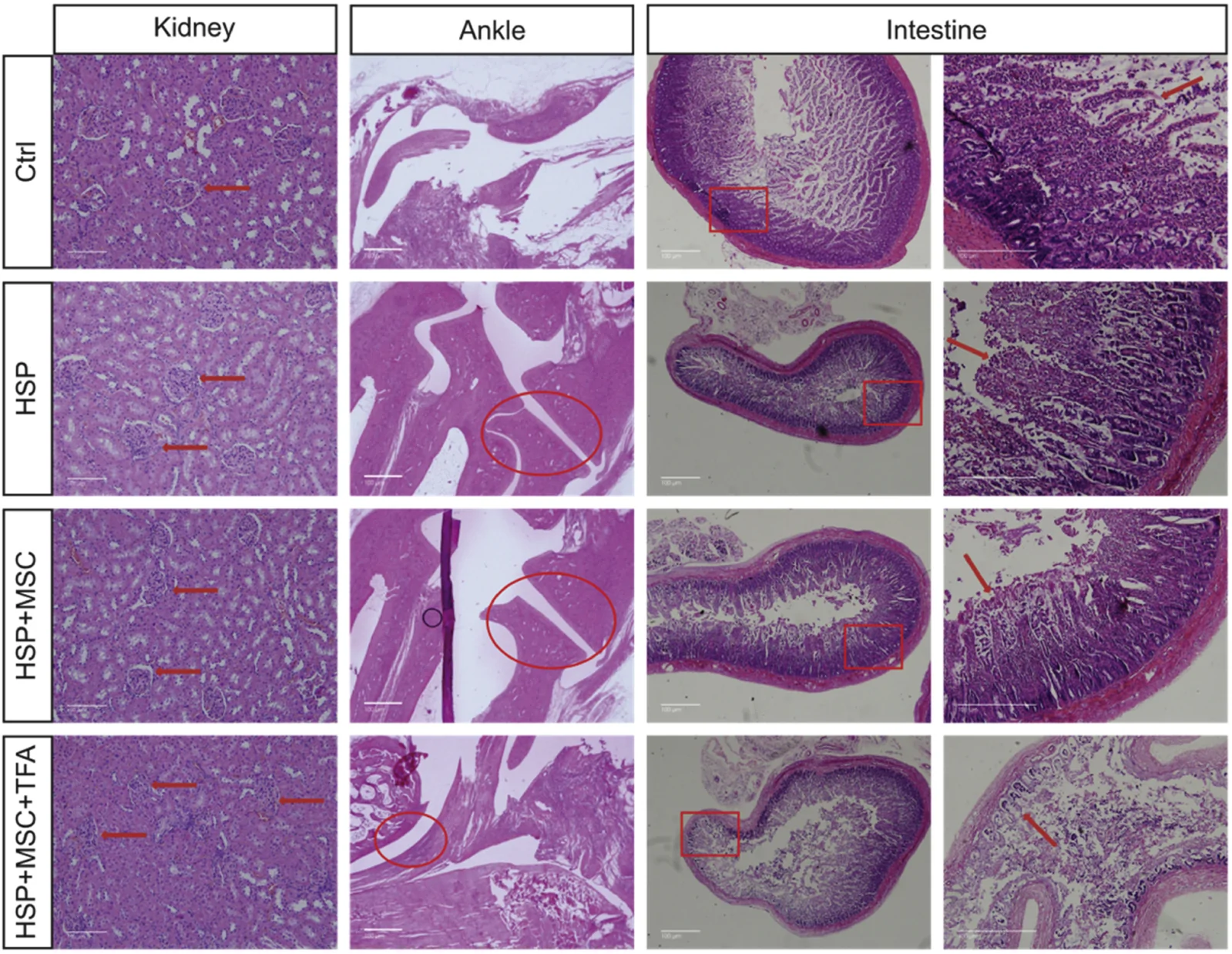
hUC-MSCs alleviate systemic pathology in an OVA-induced rat model of HSP. H&E staining of kidney, ankle joint, and intestine. HSP rats showed glomerular edema (red arrow), joint space narrowing (red circle), and villous atrophy (red rectangle and arrow). hUC-MSCs ameliorated these changes, while colivelin TFA abrogated the therapeutic effects and induced synovitis. n = 10 rats per group, Scale bar = 100 μm. Ctrl: healthy control. HSP: HSP model. HSP+MSC: hUC-MSCs treated HSP model. HSP+MSC+TFA: hUC-MSCs + colivelin TFA treated HSP model.

### hUC-MSCs restore Th17/Treg balance in HSP rats

Flow cytometric analysis of splenic CD4+ T cells revealed that HSP rats exhibited increased Th17 cells, reduced Tregs, and a significantly elevated Th17/Treg ratio (Figure 5A–C). hUC-MSCs treatment reversed this imbalance, suppressing Th17 expansion and enhancing Treg accumulation. However, STAT3 reactivation with colivelin TFA negated these effects, reinstating a Th17-dominant phenotype.

**Figure 5:**
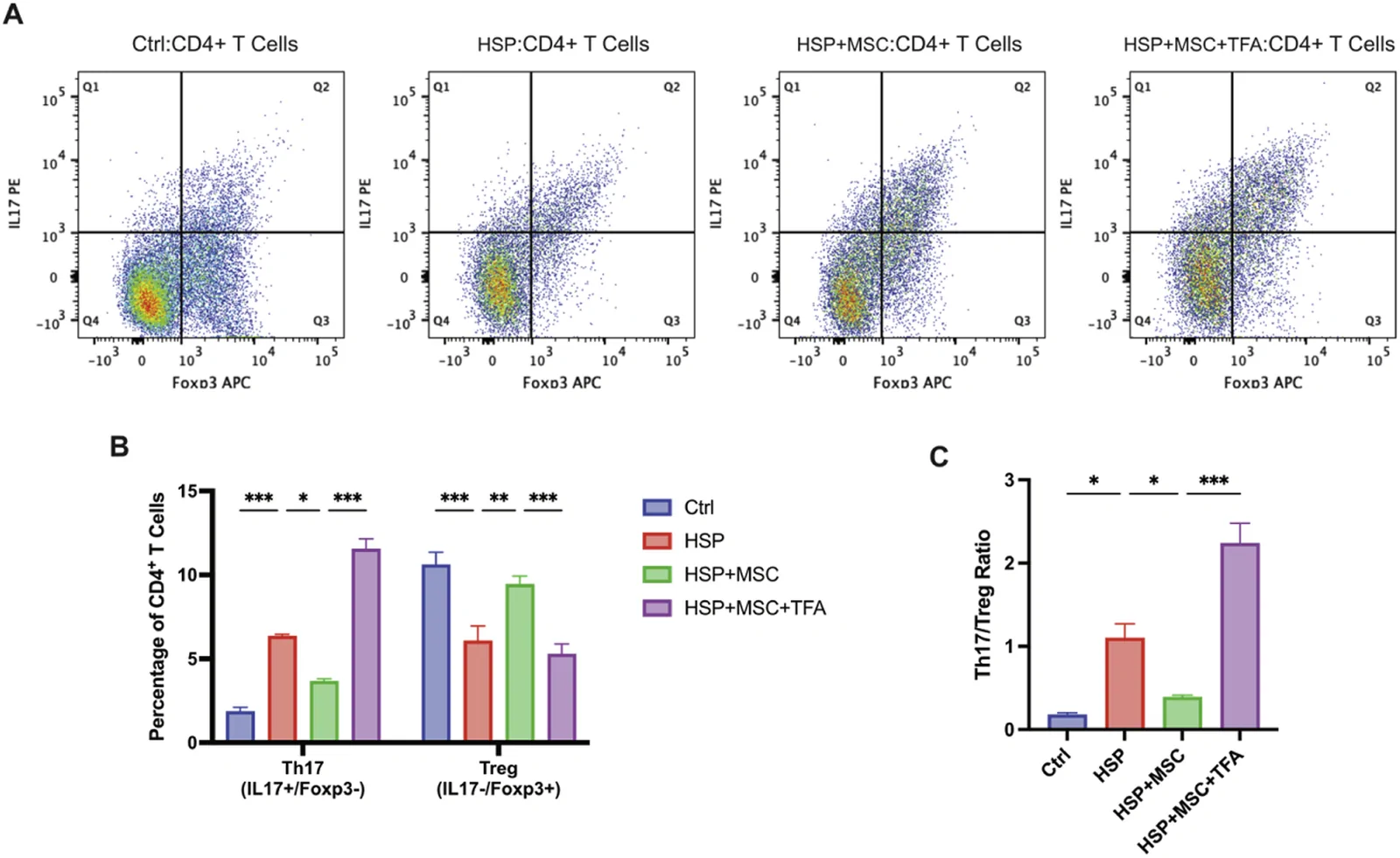
hUC-MSCs restore Th17/Treg balance in HSP rats through STAT3 inhibition. Flow cytometric analysis of splenic CD4^+^ T cells. (A) Gating strategy for Th17 (CD4^+^IL-17A^+^) and Treg (CD4^+^Foxp3^+^) subsets. (B) Percentages of Th17 and Treg cells. (C) Th17/Treg ratio across groups: healthy control (Ctrl), HSP model (HSP), hUC-MSCs treated (HSP+MSC), and hUC-MSCs + colivelin TFA treated (HSP+MSC+TFA). hUC-MSCs normalized Th17/Treg balance, whereas colivelin TFA negated this effect. n = 10 rats per group, * P < 0.05, ** P < 0.01, *** P < 0.001.

### hUC-MSCs downregulate STAT3 signaling and modulate Th17/Treg-related molecules

Western blot analysis of splenic tissues showed that HSP rats had increased expression of IL-6, STAT3, p-STAT3, and RORγt compared with controls (Figure 6A–E). These changes were accompanied by an expansion of Th17 cells and reduction of Tregs (Figure 6F–G). hUC-MSCs treatment significantly attenuated STAT3 pathway activation, suppressed RORγt, and restored Th17/Treg balance. In contrast, colivelin TFA reactivation reversed these effects, re-establishing Th17 predominance.

**Figure 6:**
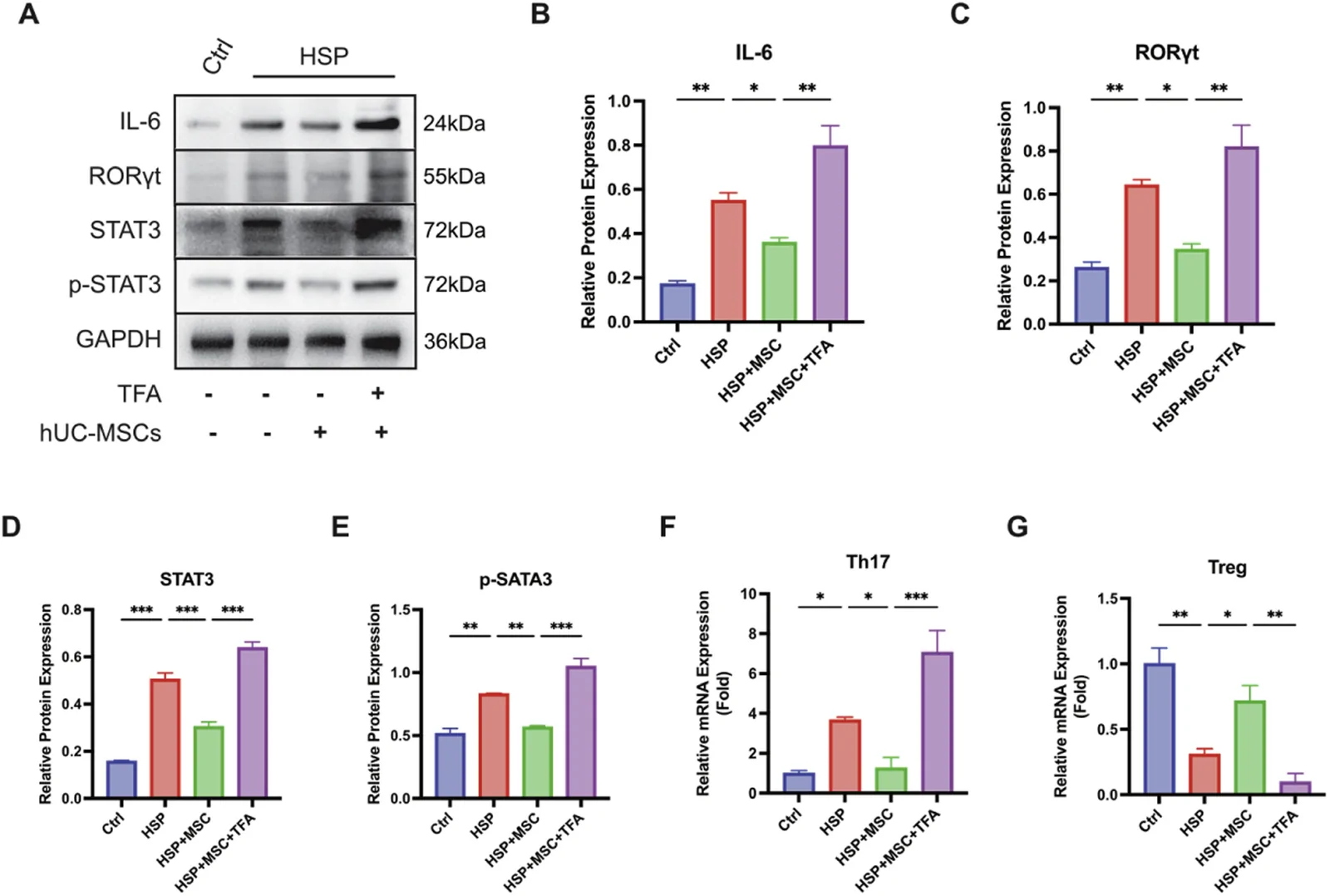
hUC-MSCs downregulate STAT3 signaling and re-establish Th17/Treg homeostasis. (A)Western blot of IL-6, STAT3, p-STAT3, and RORγt in spleen tissues from Ctrl, HSP, HSP+MSC, and HSP+MSC+TFA groups. (B–E) Quantification of IL-6, RORγt, STAT3, and p-STAT3 expression. (F–G) Relative mRNA expression of Th17- and Treg-associated markers. hUC-MSCs suppressed STAT3 activation and RORγt expression, reducing Th17 expansion and partially restoring Treg levels. Colivelin TFA reversed these effects. Data are mean ± SEM, n = 3 independent experiments. * p < 0.05, ** p < 0.01, *** p < 0.001.

## Discussion

HSP pathogenesis is multifactorial, with IgA deposition, complement activation, and endothelial injury contributing to disease [[1], [2], [3], [4]]. However, these processes alone do not fully explain clinical heterogeneity, pointing to immune dysregulation as a contributing factor.

Mechanistically, IgA1-dominant immune complexes deposited in small vessel walls trigger STAT3 activation through multiple interconnected pathways: cross-linking of FcαRI (CD89) initiates signaling cascades; complement-generated anaphylatoxins (C3a, C5a) amplify IL-6 production; and IL-6 engagement of the gp130/JAK2 complex directly phosphorylates STAT3. This IL-6/STAT3/RORγt axis promotes Th17 differentiation while suppressing Treg stability, ultimately driving endothelial activation, upregulating adhesion molecules, and increasing vascular permeability — the pathological basis of purpuric lesions. The present observation that HSP serum markedly upregulated IL-6 and p-STAT3 in both endothelial and CD4⁺ T cells (Figure 3) is fully consistent with this model, positioning STAT3 as a central node linking immune complex deposition to downstream immunopathology.

Consistent with this STAT3-driven model, the authors found a pronounced Th17/Treg imbalance in children with HSP, characterized by increased RORγt expression and IL-17 production together with reduced FOXP3 and IL-10 levels. These findings indicate that a Th17-skewed immune response characterizes pediatric HSP, and are supported by previous clinical reports and meta-analyses describing Th17/Treg imbalance in HSP and related conditions [[5], [6], [7]]. Adaptive immune dysregulation therefore represents a relevant component of HSP pathology rather than a secondary epiphenomenon.

Th17 cells, through IL-17 production, promote neutrophil recruitment and activation, leading to the release of proteolytic enzymes and reactive oxygen species that damage the endothelial lining. This IL-17-driven endothelial injury increases vascular permeability, enabling extravasation of erythrocytes into the dermis and producing the characteristic non-blanchable purpura. Conversely, Treg-derived IL-10 and TGF-β maintain endothelial barrier integrity by suppressing inflammatory cytokine production and promoting repair. The Th17-skewed imbalance the authors observed in HSP patients (Figure 1E–F) thus shifts the vascular microenvironment toward a pro-inflammatory, pro-permeability state that directly facilitates purpuric lesion development.

The present data further implicate STAT3 signaling as a potential molecular axis associated with this immune imbalance. *In vitro*, exposure of endothelial cells to HSP serum enhanced IL-6 expression and STAT3 phosphorylation, coinciding with impaired endothelial reparative capacity. Co-culture with hUC-MSCs reduced STAT3 phosphorylation and partially normalized endothelial function, and pharmacological reactivation of STAT3 using colivelin TFA attenuated these protective effects. These findings align with evidence that STAT3 activity favors Th17 differentiation while constraining Treg stability [16,17].

Given that the Transwell system excluded direct cell–cell contact, the authors attribute these effects to paracrine signaling. This is consistent with the view that MSCs exert immunomodulation primarily through secreted factors and extracellular vesicles. Known mediators include IL-10, TGF-β, and PGE₂, and extracellular vesicle-associated microRNAs such as miR-146a, which can target STAT3 signaling and reinforce FOXP3 expression [8,11]. MSCs may also shift macrophages toward an M2 phenotype, further promoting a regulatory milieu. Thus, the paracrine secretome likely constitutes the primary mechanism through which hUC-MSCs suppress the IL-6/STAT3 axis and restore Th17/Treg homeostasis in HSP, although individual mediators were not directly interrogated in the present study.

Notably, the authors observed cell type–specific regulation of STAT3 signaling. While HSP serum exposure was associated with increased STAT3 activation in endothelial cells and CD4⁺ T cells, hUC-MSCs themselves did not exhibit detectable activation of STAT3 or phosphorylation of STAT3 under comparable conditions, instead showing a selective increase in IL-6 expression. This dissociation suggests that hUC-MSCs may primarily influence STAT3 activity in neighboring cells via paracrine mechanisms rather than acting as STAT3-responsive effectors, consistent with the Transwell-based co-culture system.

In the rat HSP model, systemic inflammation was accompanied by elevated STAT3 signaling activity and a skewed Th17/Treg balance. Administration of hUC-MSCs was associated with reduced vascular inflammation, partial restoration of Th17/Treg equilibrium, and decreased activity of the IL-6/STAT3/RORγt axis. These effects were diminished by colivelin TFA, further supporting a functional link between STAT3 signaling and the immunomodulatory actions of hUC-MSCs in this context.

While STAT3 appears to be an important regulatory node, the immunomodulatory effects of MSCs are inherently pleiotropic [[18], [19], [20], [21]] involving cytokine secretion [11] extracellular vesicle–mediated microRNA transfer [22,23] and metabolic reprogramming [24]. The present data are compatible with a predominantly paracrine mode of action and provide a framework for future mechanistic dissection.

Several limitations should be acknowledged. Although no overt toxicities were observed, the safety evaluation was constrained by experimental duration and sample size; long-term adverse effects and transplantation-related risks require systematic assessment in extended studies. Pharmacological reactivation of STAT3 with colivelin TFA supports the involvement of this pathway but does not exclude potential off-target effects, and genetic approaches would provide more definitive causal evidence. In addition, the experimental models and sample sizes may not fully recapitulate human HSP complexity, underscoring the need for validation in larger cohorts and complementary model systems.

From a translational perspective, current therapeutic strategies such as corticosteroids and IVIg primarily target downstream inflammatory pathways. Corticosteroids can transiently modulate the Th17/Treg axis by inhibiting STAT3 phosphorylation, but this effect is dose-limiting, globally immunosuppressive, and often fails to sustain long-term immune homeostasis. IVIg, reserved for refractory cases, similarly does not fundamentally correct the underlying Th17/Treg imbalance. In contrast, hUC-MSCs restore Th17/Treg equilibrium through paracrine-mediated suppression of the IL-6/STAT3 axis without broadly ablating effector immune populations. Derived from routinely discarded umbilical cord tissue, they possess low immunogenicity, strong proliferative capacity, and entail minimal ethical concerns, making them an attractive candidate for pediatric applications. By targeting upstream immune dysregulation, hUC-MSCs represent a potentially disease-modifying strategy distinct from conventional symptomatic therapies. A direct comparison between hUC-MSCs and IVIg regarding STAT3 signaling and Th17/Treg modulation in HSP is a key direction for future investigation; the authors hypothesize that these modalities may converge on STAT3 through distinct pathways.

In summary, this study suggests that hUC-MSCs modulate STAT3 signaling, protect endothelium, and rebalance Th17/Treg in experimental HSP. While STAT3 is not the sole mechanism, its central position in immune-endothelial networks highlights its therapeutic relevance, providing a basis for future translational studies.

## Conclusion

This study identifies Th17/Treg imbalance as a central immunopathological feature of HSP, with elevated RORγt/IL-17 and reduced FOXP3/IL-10. hUC-MSCs restore this balance by suppressing STAT3 phosphorylation, downregulating IL-6, inhibiting Th17 expansion, and promoting Treg stability. *In vitro*, hUC-MSCs protected endothelial cells; *in vivo*, they alleviated multi-organ inflammation. Compared with conventional therapies that provide transient symptomatic relief, hUC-MSCs offer a mechanistically distinct, disease-modifying approach with favorable accessibility and safety profiles. Crucially, these effects were abrogated by pharmacological reactivation of STAT3, confirming the pathway as a key therapeutic target. These findings not only provide mechanistic insight into HSP pathogenesis but also support the development of hUC-MSC-based therapies as a promising and targeted strategy for pediatric vasculitis.

## Authors' contributions

Li Zhang: Conceptualization, Investigation, Formal analysis, Writing – Original Draft. Tao Chen: Methodology, Investigation, Data Curation, Writing – Original Draft. Jieqiong Zeng: Resources (patient cohort management), Validation. Anna Qiao: Investigation, Visualization. Xianjuan Shen: Resources (laboratory assays and diagnostics), Supervision. Zhiyuan Tang: Resources (pharmacological analysis and materials), Writing – Review & Editing. Jianmei Zhao: Supervision, Project administration, Funding acquisition, Writing – Review & Editing. All authors critically reviewed and approved the final manuscript.

## Funding

This work was supported by the General Program of National Natural Science Foundation of China (82270528), the General Program of Nantong Municipal Health Commission (MS2024005), the 6th Jianghai Talent Cultivation Program of Nantong City, the Jiangsu Medical Association Project Research (SYH-32034-0144), and the 14th Five-Year Plan Sci-Edu Strengthening Healthcare Project of Nantong Municipal Health Commission (NTCXTD43).

## Conflicts of interest

The authors declare that they comply with the PCI rule of having no financial conflicts of interest in relation to the content of the article.

## Data Availability

All data generated or analyzed during this study are included in this published article and its supplementary information files.

## Appendix Supplementary materials

Image, application 1
