# Supplementary material for: hUC-MSCs regulating the balance of Th17/Treg in Henoch Schönlein purpura via STAT3 pathway
Source: J Pediatr (Rio J). 2026 Oct 13;102(6):101613. doi: 10.1016/j.jped.2026.101613 (PMC13685973; doi:10.1016/j.jped.2026.101613)
Supplement: Supplementary file 1 [file mmc1.docx]

**JPED-D-26-00086**

## Supplementary Material

Note: (A) Experimental schematic. (B–E) Representative images of skin lesions: (B) healthy control (Ctrl), (C) HSP model (HSP), (D) hUC-MSCs treated (HSP+MSC), and (E) hUC-MSCs + colivelin TFA treated (HSP+MSC+TFA).
